# In Vivo Study of Entero- and Hepatotoxicity of Silver Nanoparticles Stabilized with Benzyldimethyl-[3-myristoylamine)-propyl]ammonium Chloride (Miramistin) to CBF1 Mice upon Enteral Administration

**DOI:** 10.3390/nano11020332

**Published:** 2021-01-27

**Authors:** Yurii A. Krutyakov, Alexey A. Kudrinskiy, Vladimir A. Kuzmin, Jaeho Pyee, Alexander A. Gusev, Inna A. Vasyukova, Olga V. Zakharova, Georgy V. Lisichkin

**Affiliations:** 1 Department of Chemistry, Lomonosov Moscow State University, 1-3 Lenin Hills, 119991 Moscow, Russia; akudrinskiy@yandex.ru (A.A.K.); lisich@petrol.chem.msu.ru (G.V.L.); 2 National Research Center “Kurchatov Institute”, Akademika Kurchatova pl. 1, 123182 Moscow, Russia; 3 V. P. Urban Department of Epizootology, Saint-Petersburg State University of Veterinary Medicine, 5 Chernigovskaya st., 196084 St. Petersburg, Russia; kuzmin@epizoo.ru; 4 Department of Molecular Biology, Dankook University, 119 Dandae str., Cheonan 31116, Korea; jpyee1@dankook.ac.kr; 5 Technopark “Derzhavinsky” Derzhavin Tambov State University, 33 Internatsionalnaya st., 392000 Tambov, Russia; nanosecurity@mail.ru (A.A.G.); vasyukovaia@gmail.com (I.A.V.); olgazakharova1@mail.ru (O.V.Z.); 6 Department of Functional Nanosystems and High-Temperature Materials, National University of Science and Technology MISiS, Leninskiy prospekt 4, 119049 Moscow, Russia; 7 Engineering Center, Plekhanov Russian University of Economics, Stremyanny Lane 36, 117997 Moscow, Russia

**Keywords:** silver nanoparticles, benzyldimethyl[3-myristoylamine)-propyl]ammonium chloride monohydrate, enterotoxicity, hepatotoxicity, acute experiment, subacute experiment

## Abstract

Silver nanoparticles (AgNPs) are the most widely studied antimicrobial nanomaterials. However, their use in biomedicine is currently limited due to the availability of data that prove the nanosilver toxicity associated primarily with oxidative stress development in mammalian cells. The surface modification of AgNPs is a potent technique of improvement of their biocompatibility. The synthetic or natural compounds that combine zero or low toxicity towards human and animal organisms with inherent antimicrobial properties are the most promising stabilizing agents, their use would also minimize the risks of microorganisms developing resistance to silver-based materials. We used a simple technique to obtain 30–60 nm AgNPs stabilized with benzyldimethyl[3-myristoylamine)-propyl]ammonium chloride monohydrate (BAC)—a well-known active ingredient of many antibacterial drugs. The objective of the study was to assess the AgNPs-BAC entero- and hepatotoxicity to CBF1 mice upon enteral administration. The animals were exposed to 0.8–7.5 mg/kg doses of AgNPs-BAC in the acute and to 0.05–2.25 mg/kg doses of AgNPs-BAC in the subacute experiments. No significant entero- and hepatotoxic effects following a single exposure to doses smaller than 4 mg/kg were detected. Repeated exposure to the doses of AgNPs-BAC below 0.45 mg/kg and to the doses of BAC below 0.5 mg/kg upon enteral administration also led to no adverse effects. During the acute experiment, the higher AgNPs-BAC dose resulted in increased quantities of aminotransferases and urea, as well as the albumin-globulin ratio shift, which are indicative of inflammatory processes. Besides, the relative mass of the liver of mice was smaller compared to the control. During the subacute experiment, the groups treated with the 0.25–2.25 mg/kg dose of AgNPs-BAC had a lower weight gain rate compared to the control, while the groups treated with the 2.25 mg/kg dose of AgNPs-BAC showed statistically significant variations in the blood serum transaminases activity, which indicated hepatosis. It should be noted that the spleen and liver of the animals from the groups treated with the 0.45 and 2.25 mg/kg dose of AgNPs-BAC were more than two times smaller compared to the control. In the intestines of some animals from the group treated with the 2.25 mg/kg dose of AgNPs-BAC small areas of hyperemia and enlarged Peyer’s patches were observed. Histological examination confirmed the initial stages of the liver and intestinal wall inflammation.

## 1. Introduction

Silver is the sixty third most common element in the Earth’s crust and silver compounds can be found on every continent. It is hard to say when people first discovered silver, but it is clear that it was known and used by prehistoric people. Silver alloys were actively used for producing coins, ornaments and various household items. The medical properties of the metal, especially its powerful antibacterial effects, were known to people at the time of Hippocrates. Later, there were attempts to use silver for treatment of epilepsy, neonatal ophthalmic diseases, cholera, dysentery, sexually transmitted diseases, traumatic and other types of infections. One should also mention surgical instruments, bone prostheses and catheters made of silver alloys [1,2]. Developments in biotechnology have allowed incorporation of ionizable silver into fabrics for clinical use to reduce the risk of nosocomial infections, and for personal hygiene [3,4].

Discovery of colloidal silver at the end of the 19th century, when the German surgeon and the son of C. Credé (a famous gynecologist who introduced silver nitrate eyedrops as an antiseptic for the prevention of ophthalmia neonatorum in newborns)—B. Credé (1847–1929) together with several chemists suggested using nonionized silver in the form of colloidal solutions of metallic silver (“Unguentum Credé”, collargol) or silver oxide sol, which can be considered a breakthrough in the medical and pharmaceutical application of the metal [5]. Outstanding antimicrobial activity together with the lack of resistance of most pathogens make silver a very promising material for medical applications especially taking into account the new life-threatening antibiotic-resistant bacterial strains [4].

Development of nanoindustry gave rise to vast research of silver nanoparticles (AgNPs). Active studies are being conducted in order to receive a better understanding of both existing and potential application of AgNPs for water purification and disinfection [6,7], clothing and footwear manufacturing [8], cosmetics [9], household appliances and cleaning products, etc [10,11]. The exceptional potential of AgNPs for application in medicine and hygiene practices can be explained through their prominent antibacterial [12,13], antiviral [14] and antifungal [15,16] properties. Currently, AgNPs-based wound dressing materials and water purifying agents are also being actively developed [4,17].

Though silver has been actively used throughout most of human history, the first data on its potential toxic effects only appeared in the 20th century [4]. AgNPs are the least studied silver-containing substances, and the data on their toxicity to mammals and humans are inconclusive and often contradictory. Upon oral AgNPs administration, the first pathological changes are observed in the digestive tract and liver [18,19]. It is a well-known fact that a healthy human gastrointestinal tract is colonized by about one trillion commensal bacteria comprising over 3000 species that form intestinal microbiota [20]. The intestinal microbiome is of utmost importance to human health while any imbalance can affect the host’s metabolism and immunity which are closely connected with chronic inflammatory diseases [21]. It is reasonable to assume that when administered orally, AgNPs might damage the intestinal microbiota balance due to their powerful antibacterial effects. The results of a study where AgNPs stabilized with polyvinylpyrrolidone (PVP) with a diameter of 10–80 nm were orally administered to rats for 62 days in doses of 0.1 mg/kg, 1.0 mg/kg and 10 mg/kg of body weight per day indicated that AgNPs produced no significant impact on the composition of the main components of normal rat cecum microbiota. The total amount of aerobe and anaerobe bacteria, the volume of lactobacilli and enterobacteria remained unchanged in all studied groups. At the same time, the bifidobacteria population inhabiting the bodies of rats receiving the 0.1 mg/kg dose of AgNPs was reduced compared to the control group, although the absolute value of the decrease was not significant (less than half of the initial quantity). Besides, for streptococci and staphylococci in the group receiving the 10 mg/kg dose of AgNPs, the total values were below those of the control group by more than an order of magnitude [22]. Alkaline Comet Assay and Micronucleus Assay were used to study the effects of orally administered 20 nm AgNPs at a dose of 50 mg/kg, 150 mg/kg and 300 mg/kg on male and female mice. Comet assay was performed on liver, spleen, blood, duodenum and kidney, and Micronucleus assay was performed on spleen lymphocytes, to evaluate the genotoxic potential. The result displayed that AgNPs first accumulated in duodenum from where they migrated to kidney, liver and spleen; in liver and duodenum cells, the nanoparticles (NPs) were discovered both in cytoplasm and in organelles, except the cell nucleus. However, the authors discovered neither genotoxicity, nor tissue damage in all the studied organs [23]. Jeong et al. [24] showed that the rats treated for 28 days with 60 nm AgNPs at a dose of 30 mg/kg, 300 mg/kg and 1000 mg/kg exhibited higher numbers of goblet cells, resulting in more mucus materials in the intestinal canal.

After entering blood vessels, AgNPs are mostly accumulated in the liver [25,26], often leading to pathological changes in its tissues and cells. In order to study the pathological potential of 8.7 nm AgNPs, the NPs were administered abdominally to female rats for 28 days at a dose of 1 mg/kg, 2 mg/kg and 4 mg/kg. Identification of the oxidative stress signs in the liver tissue, determination of the silver nanoparticles concentration in tissues, description of hepatic histopathological alterations and detection of possible chromosomal aberrations in the bone marrow were carried out. Results revealed various dose-dependent hepatic histopathological lesions. The effect of AgNPs on the hepatic malondialdehyde and glutathione levels varied in different treated groups compared to the control. Residues of AgNPs were found in the hepatic tissue and their amount depended on the initial treatment dose. In addition, AgNPs induced variable chromosomal aberrations that were dose dependent. The reported results display the hepato- and genotoxic properties of AgNPs [27]. A single intravenous exposure of rats to 20.1 ± 2.8 nm AgNPs (5 mg/kg) produced no toxic effects on the liver cells despite considerable AgNPs accumulation in the liver (60% of the introduced NPs within the first 24 h) according to the histopathological and biochemical studies [25]. Park et al. [28] studied the effects of the exposure of mice 42 nm AgNPs at a dose of 0.25 mg/kg, 0.5 mg/kg and 1 mg/kg for 28 days. The authors observed a dose-dependent increase of the alanine transaminase (ALT) and aspartate transaminase (AST) levels in all treated groups. Although the histological studies of the liver and intestine tissues revealed no pathologies, the adverse impact on liver and kidney was observed in the high dose-treated group (1.00 mg/kg). In the longer lasting experiment where the doses of 0.1 mg/kg, 1 mg/kg and 10 mg/kg of PVP-stabilized AgNPs were administered to rats for 92 days, N.V. Zaytseva et al. [29] observed histological changes in the liver tissue even in the case of the lowest studied dose. Eosinophilic infiltration of the portal tracts was also observed, accompanied by the emergence of medium and large-drop fat vacuoles in the cytoplasm of hepatocytes, swelling and lympho-macrophage infiltration of the portal tracts at the doses of 1.0 and 10.0 mg/kg. These changes can be treated as symptoms of inflammation of hepatocytes.

Interestingly, in some cases, the higher doses of AgNPs produce a less pronounced toxic effect, as shown, for example, in the paper by Kim et al. [30]. Their research revealed that when exposed to orally administered 60 nm AgNPs at the doses of 30 mg/kg, 300 mg/kg and 1000 mg/kg, rats did not show any significant changes in the body weight that could be related to the doses of AgNPs during the 28-day experiment. In the case of the maximal dose, biochemical blood examination revealed some significant changes in the alkaline phosphatase and cholesterol levels related to bile ducts hyperplasia revealed during histological examinations.

In the 14-day experiment on BALB/C mice receiving 20 and 50 ppm of 40 nm AgNPs orally, the researchers observed significantly increased levels of ALT and AST. During histological examination hepatotoxic effects in the form of necrosis, hepatocytic inflammation and total lymphocyte aggregation were observed [31]. Similar results were obtained when 30–50 nm AgNPs were orally delivered to rats for 21 days at a dose of 50 mg/kg. The treatment resulted in severe hepatotoxicity and histological changes, increased DNA fragmentation and down regulation of the antioxidant gene expression in the liver [32].

Intraperitoneal administration of 33 nm AgNPs at a dose of 78 mg/kg to mice during the period of 14 days resulted in the pronounced increase in the ALT (+126.6%) and AST (+53.5%) levels, while the histological inspection of the liver revealed vascular hyperemia as well as parenchymatous and perivascular aggregations of mononuclear cells [33]. Another 30-day study where rats were injected intraperitoneally three times a week with 54.9 nm AgNPs revealed a severe hepatotoxic effect. AgNP exposure enhanced the hepatic lipid peroxidation (+281.7%) along with a decline in the reduced glutathione (−58.3%) levels. The apparent hepatic oxidative damage was associated with the obvious hepatic dysfunction as shown by the alteration of the serum liver enzymatic biomarkers, the lipid profile and the pathological hepatic lesions. Variously located cases of focal liver necrosis with inflammatory cell infiltration, hydropic cell degeneration and bile duct epithelium degeneration were observed [34].

Various methods of the AgNPs’ toxicity reduction are currently being studied, such as green synthesis [35,36] or NPs stabilization and surface functionalization [37]. In the paper [37], the authors investigated the hepatotoxicity of citrate-coated AgNPs (cAgNPs) after single intravenous administration to rabbits at the doses of 0.5 mg/kg and 5 mg/kg. The results showed that the structure and function of the liver tissue were disrupted. The authors confirmed the development of dose-dependent oxidative stress in the liver tissue significantly exceeding the control group levels. It should be noted that the levels of oxidative stress markers remained abnormal 28 days after the single exposure to cAgNPs, suggesting a potential genotoxic effect of the studied nanoparticles. Toxicity of cAgNPs was evaluated in a longer lasting (42 days) experiment where rats received the 62.5 mg/kg, 125 mg/kg and 250 mg/kg doses of the material orally. Hematologic study, biochemical blood serum analysis and histopathological examination revealed no significant differences between the treated and the control groups. Toxicity endpoints of the reproduction screening test were measured and revealed no evidence of the cAgNPs toxicity [38].

Oral administration of the doses of 50 mg/kg, 100 mg/kgand 200 mg/kg of PVP-covered AgNPs to rats for 90 days led to a dramatic increase in reactive oxygen intermediate production that, as a result, stimulated a dose-dependent increase in the hepatotoxicity markers, intensified autophagy and depleted the insulin signaling pathways. Such effects can influence apoptotic, necrotic and autophagic molecular processes [39].

Thus, finding stabilizers for AgNPs that would increase their biocompatibility remains an important problem. The goal of our research was to modify the surface of AgNPs in a way that would reduce their potential toxicity to organs and cells of mammals without compromising the antibacterial effectiveness of nanosilver.

Previously we have studied the in vitro activity of AgNPs stabilized with benzyldimethyl[3-myristoylamine)-propyl]ammonium chloride monohydrate (BAC) against gram-positive and gram-negative bacteria, yeast and fungi. BAC is a well-known active ingredient used in many antiseptic medicines. It was assumed that modification of the AgNPs surface with a cationic surfactant displaying no toxicity against mammal cells [40,41], and possessing antibacterial properties [42,43,44,45] would produce a highly effective antibacterial agent containing small-diameter AgNPs with the positive surface charge that would require no additional surface modification or purification [46,47,48,49]. During in vitro experiments we confirmed high effectiveness of AgNPs-BAC. In particular, its antibacterial activity against E. coli was twice higher than that of unmodified AgNPs and twenty times higher than the activity displayed by BAC, making this combination a powerful antibacterial agent [50]. This effectiveness was confirmed in in vivo experiments when it was used for treating enteritis of dogs [51]. Stabilization with BAC may reduce the toxic effects of unmodified AgNPs. Thus, the present work is aimed at evaluating the entero- and hepatotoxicity of the AgNPs-BAC active complex by means of acute and sub-acute in vivo experiments on laboratory mice.

## 2. Materials and Methods

AgNPs were synthesized according to the method described in our previous work [52]. Benzyldimethyl[3-myristoylamine)-propyl]ammonium chloride monohydrate (BAC)—trade name Miramistin^®^ (Infamed K, LLC, Kaliningrad Oblast, Russia)—a commercially manufactured antiseptic medicine, belongs to the chemical group of quaternary ammonium compounds efficient against pathogenic microorganisms including fungi, bacteria and protozoa. In order to study the effects of BAC on animals, the substance was administered at a dose of 0.5 mg/kg.

### 2.1. AgNPs-BAC

AgNPs-BAC were obtained via the Tollens’ technique by reducing the ammonia silver complex with glucose during gentle heating. The process consisted of three stages:

(a) Obtaining an ammonia complex of silver oxide:

150 µL of 0.01% sodium hydroxide solution (3.8 × 10^−5^ mol) were added to 50 mL of aqueous solution of 0.017 g (1 × 10^−4^ mol) of silver nitrate. Then, the mixture containing unreacted silver nitrate and precipitated silver oxide was mixed with a 25% ammonia solution (approx. 50 μL) until full dissolution of Ag_2_O was achieved.

(b) Reducing diamminesilver(I) complex with glucose in the presence of BAC as a capping agent:

50 mL of an aqueous solution of 0.011 g of BAC were added dropwise to the obtained diamminesilver(I) complex. After 15 min of stirring, 0.35 g of glucose were added. Reduction process took place at 49 °C.

(c) Purification of the AgNPs’ dispersion:

Dispersion of AgNPs with the remains of reagents like glucose, sodium hydroxide and ammonia was purified by means of dialysis. Dialysis of dispersion of AgNPs was carried out by soaking of dialysis bags SERVAPOR^®^ (SERVA Electrophoresis GmbH, Heidelberg, Germany) with pore diameters of 2.5 nm containing dispersions of AgNPs-BAC into a 0.01% solution of pure BAC in double distilled water with the ratio of volumes of dispersion of silver and solution of BAC equal to 1:10. Dialysis was carried out twice in the same conditions. The obtained purified dispersions were diluted with the 0.01% solution of BAC until the content of colloidal silver in them comprised 10 and 50 ppm, respectively.

### 2.2. Transmission Electron Microscopy (TEM)

Micrographics of AgNPs were obtained using an electronic microscope (Leo 912 AB Omega, Leo Ltd., Neu-Isenburg, Germany) with the operating accelerating potential of 100 kV. In order to prepare the samples, 1–2 μL of the solution were spread onto the copper mesh coated with Formvar™ (d = 3.05 mm), which was then dried in the open air for 5–10 min. Size distribution of AgNPs was calculated on the basis of the obtained micrographs using the software Femtoscan Online v. 2.2.91 (Center of Perspective Technologies, Moscow, Russia).

### 2.3. Electron Spectroscopy

To register the absorption spectra in the visible region, a spectrophotometer (JENWAY 6310 Bibby Scientific Ltd., Stone, UK) and quartz cells with the 10 mm optical path length were used.

The aqueous solution was prepared in distilled water with the AgNPs content equivalent to the tested dosage; the AgNPs were stabilized with BAC in the ratio 1:10.

### 2.4. Zeta Potential

Zeta potential was measured by applying an electric field across the dispersion of silver NPs using the technique of laser Doppler anemometry that involved the ZetasizerNano ZS analyzer (Malvern Instruments Ltd., Malvern, UK).

### 2.5. Animals and Conditions

Six-week-old male CBF1 mice were used during the experiment (body weight 25 ± 2 g). All manipulations with the animals were carried out in accordance with the Guide for the Care and Use of Laboratory Animals (ILAR, DELS), the Principles of good laboratory practice [53]. The Saint Petersburg State University of Veterinary Medicine Institutional Animal Care and Use Committee approved the animal related procedures in this study (#011–05–19).

The animals were housed according to National Standard [54] in 440 × 270 × 140 mm^3^ polycarbonate boxes, 10 animals per box. Sterilized wood shavings were used as bedding. The following ambient conditions were kept under strict control: air temperature 20–22 °C, relative humidity 50 ± 5% and a 12:12 light–dark cycle. Throughout the experiment, the animals had unlimited access to food and water. Prior to the experiment, the animals underwent a 14-day quarantine period.

### 2.6. Experiment

Acute experiment. In order to evaluate the AgNPs-BAC acute toxicity, the mice were divided into 9 groups, 10 animals in each group. AgNPs-BAC were administered using an intragastric gavage as a single dose of 0.8; 1; 1.2; 1.5; 4; 5; 7.5 mg/kg. The AgNPs-BAC doses were selected according to the previously obtained results for non-stabilized and non-functionalized AgNPs [27,28]. The BAC group received the pure BAC solution in a dose of 5 mg/kg. The dose was selected based on the results of the previous research defining the optimal dose for AgNPs stabilization. The control group received the same volume of distilled water. The animals were closely observed for 14 days after the treatment, the functional status of the body was regularly evaluated and the animals were weighed once a week. At the end of the observation period, all animals were euthanized for further morphological, biochemical and histological examinations.

Subacute experiment. In order to evaluate the AgNPs-BAC subacute toxicity, 6 groups were formed, 15 animals in each group. 0.5 mL of the dispersion were administered daily for 14 days using an intragastric gavage. AgNPs-BAC were administered in the doses of 0.05; 0.25; 0.45; and 2.25 mg/kg. The BAC group received pure BAC solution at a dose of 0.5 mg/kg. The control group received the same volume of distilled water. Daily examinations together with evaluation of the functional status of the body were carried out. The animals were weighed once a week. The following parameters were observed: skin and coat health, body position, food and water consumption, weight gain dynamics. At the end of the observation period, all animals were euthanized for further morphological, biochemical and histological examinations.

### 2.7. Functional Status of the Animals

Activity—low, normal, increased.

Movements—loss of coordination, muscle tonus, tremor.

Body position—natural, lying on one side, hunched posture, hiding in the corner.

Appearance:

Body condition—adipose, well-conditioned, seriously underweight, cachectic.

Coat—shiny, dull, smooth, rough, abnormal shedding.

Eyes—watery eyes, inflammation, corneal clouding, adhesion.

Ears—color (normal, pale, reddened), inflammation, discharge, crusting, twitching.

Teeth—color, broken teeth, tooth loss.

Feet and limbs—color, swelling.

Physiological functions:

Breathing—rapid, normal, bradypnea, wheezing, coarse breathing, panting.

Salivation—insufficient or excessive.

Saliva—watery or sticky.

Urine—color.

Excreta—color, consistency.

Food and water consumption—normal, insufficient or excessive.

The animals were weighed once a week in order to determine the weight gain dynamics during the experiment.

### 2.8. Hematological Examinations

The blood for the analysis was collected using 1.5 mL polyethylene tubes, incubated for 4 h at 37 °C until clotted, then centrifuged for 10 min at 3000× *g*. The harvested blood serum was stored at −20 °C until tested. The biochemical analysis was performed using a MindreyBA88A spectrophotometer with Olvex kits (Russia).

For red blood cell count, the full blood aliquot was diluted 1:200 with normal saline. The assay was performed within 2–3 h of dilution. For white blood cell count, full blood was diluted with 4% acetic acid using methylene blue for cell nuclei staining. The leukogram was calculated in blood smears stained by the Romanovsky-Gimza dye.

Macroscopic examinations and internal organs morphometry.

Following the experiment the animals were euthanized and the autopsy was performed for macroscopic examination of the internal organs. The organs were weighed using a digital weighing balance AND EK-6100i. For each organ, the mass coefficient *k_m_* was calculated according to the formula:

*k_m_ = m_o_/m_b_*

where *m_o_* is the individual organ mass and *m_b_* is the body mass.

### 2.9. Internal Organs Histology

The liver and intestine were fixated in a 10% buffered formalin solution, prepared and embedded in Histomix after the standard procedures [55]. The embedded material was sectioned into slices of 4 μm; the slides were stained with haematoxylin and eosin.

For the intestine histological examination, a section of the jejunum 10–11 cm from the stomach was taken. Each fragment yielded 10–15 slices from three different areas. Three randomly selected samples from each animal were photographed. In each Section, 10 fields of 130,834 μm^2^ (418 μm × 313 μm) were randomly selected and photographed. The fields included all jejunum wall layers.

In the intestinal crypts, the percentage of cells with mitotic figures was calculated. The experimental and control groups were compared on the basis of this parameter. The cells in mitotic prophase were excluded from the total of cells with mitotic figures. The non-dividing cells were calculated by their nuclei. We counted the cells in the middle part of each crypt (about 50 μm from the bottom and from the opening of the crypt). Over 1000 crypt cells were counted for each animal.

For the histological liver examination, two sections from the median lobe were taken. From each sample, at least 10 sections were prepared. Three randomly selected samples from each animal were microphotographed. The fields were randomly selected. The microphotograph area corresponded to the area of the section (130,834 μm^2^, 418 μm × 313 μm).

The number of Kupffer cells was counted for each microphotograph, then the number of cells per 1000 μm^2^ of the liver parenchyma was calculated. The cells were determined by the nuclei. The quantities for the experimental and control groups were then compared.

### 2.10. Statistical Analysis

The statistical significance of differences between the mean values was tested using Student’s *t*-test, Mann-Whitney U-test and ANOVA. *p* values < 0.05 were considered statistically significant.

## 3. Results

### 3.1. Obtaining of AgNPs-BAC

**Figure 1:**
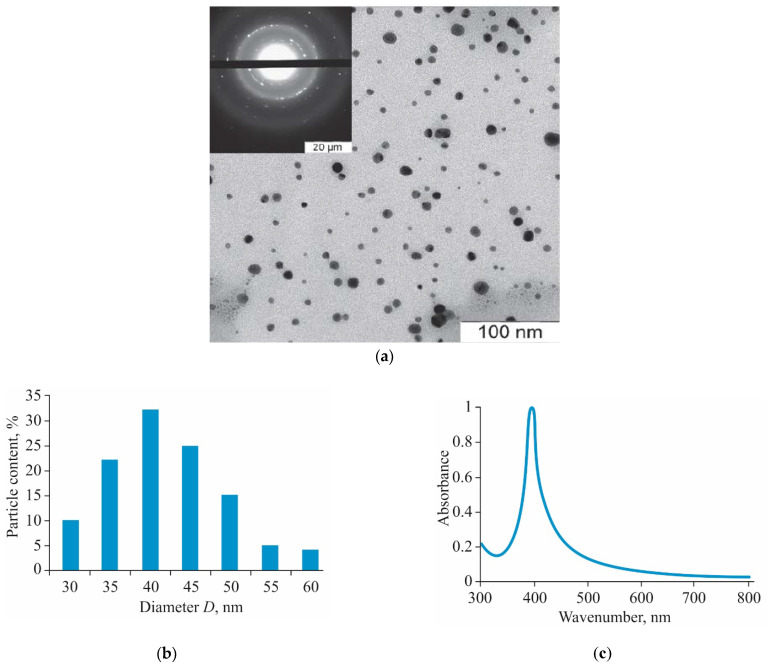
TEM image of silver nanoparticles (AgNPs) (**a**), size distribution histogram (**b**) and UV-visible absorbance spectrum (**c**) of AgNPs stabilized with benzyldimethyl[3-myristoylamine)-propyl]ammonium chloride monohydrate (AgNPs-BAC).

The obtained AgNPs-BAC is an aqueous dispersion of AgNPs capped with the biologically active cationic surfactant benzyldimethyl[3-(miristoylamino)-propyl]ammonium chloride, which is an active ingredient of many antiseptic drugs. In Figure 1a electronic micrographs are presented, confirming the presence of spherical AgNPs with a diameter of 30–60 nm, the average diameter being 40 nm (Figure 1b), in the obtained composition. The results of the electronic microdiffraction show that NPs consist of crystal metallic silver.

AgNPs-BAC is a dispersion of yellow color with a red–orange shade with pH 7.0–8.0. The ultraviolet-visible (UV-Vis) absorption spectrum (Figure 1c) of AgNPs-BAC has a characteristic narrow peak of surface plasmon resonance of AgNPs within the range of 400–413 nm.

**Figure 2:**
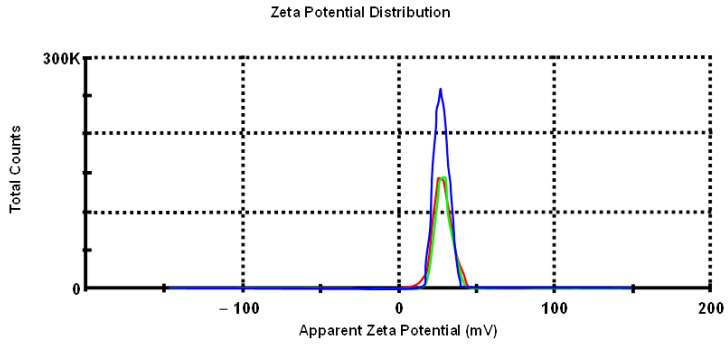
Zeta potential measurements (st. dev. −4.34).

To estimate the colloidal stability of the obtained dispersions, the zeta potential (zeta) of the NP sols was evaluated. In general, the absolute value of the zeta potential can be treated as an indicator of the stability of a colloidal system. The higher the absolute value, the higher the potential difference between the dispersion medium and the slipping plane, the higher the charge of the particles’ surface and, therefore, the larger the electrostatic repulsion between particles. As Figure 2 shows, the average zeta potential was 27.9 mV.

### 3.2. Acute Experiment

#### 3.2.1. Functional State of the Animals

The experiment showed that none of the doses of the aqueous solutions of AgNPs-BAC and BAC were lethal to the animals. As all tested doses were absolutely tolerable, we were unable to calculate the LD_50_ value.

After the administration procedure, the animals displayed anxiety and increased motion activity that lasted for 5–10 min; after that, the animals calmed down and behaved normally.

Daily external examination of the mice showed that all animals had normal physique. The body position was normal, and the behavior and reaction to the external stimuli were adequate. The feet and limbs showed no swelling or deformation. The coats were smooth and shiny, without any signs of scratching or ulceration, and no bald or scaly patches were observed. All tested animals had healthy skin with a moderately developed subcutaneous fat layer. The teeth were undamaged. All mice had healthy-looking mucous membranes. The external examination revealed no abnormal discharge from the natural orifices.

**Figure 3:**
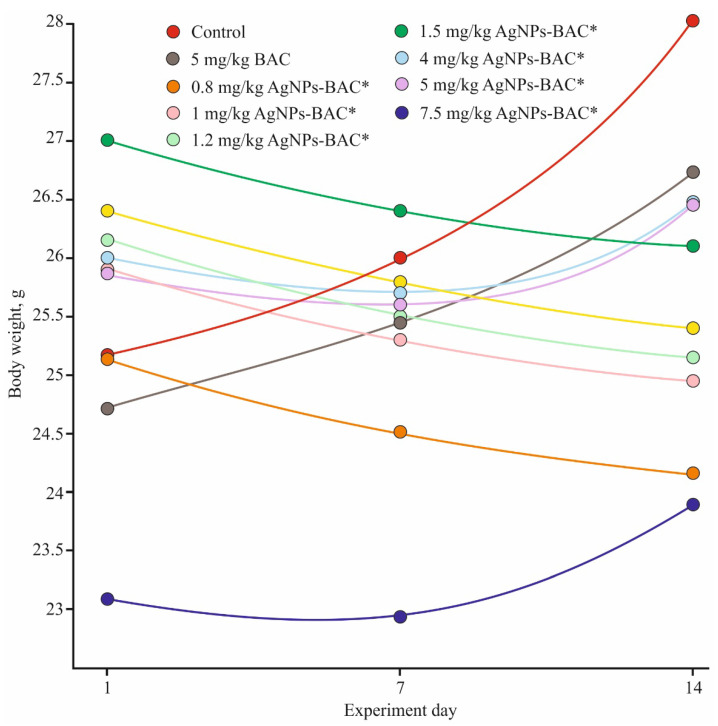
Weight gain dynamics in the acute experiment. * *p* < 0.01.

The body weight of all animals from the control and the BAC groups was strictly positive throughout the experiment. In all AgNPs-BAC-treated groups, the body mass values varied from the control, but the pattern of change differed from group to group. The groups receiving the 0.8–1.5 mg/kg dose displayed negative dynamics that might be explained by the reaction of the organism to a foreign substance. In the groups receiving the 4 mg/kg, 5 mg/kg and 7.5 mg/kg doses, the body mass of the animals decreased during the first seven days, while during the second week of the experiment we observed a consistent weight gain (Figure 3).

#### 3.2.2. Biochemical and Hematological Examinations

Exposure to the 0.8–4 mg/kg dose of AgNPs-BAC had no adverse effects on the homeostasis and the hematopoietic system and produced no significant alterations in the biochemical parameters. At the same time, in the groups receiving the 5 mg/kg and 7.5 mg/kg doses, we registered an increase in the aminotransferase content in the blood serum due to hepatolysis or hepatitis caused by the studied material. The tests also revealed a shift in the albumin–globulin ratio resulting either from the excessive consumption of albumin as the primary toxin-binding protein, or from the increase in the globulins production caused by inflammation. The increased protein utilization was also revealed as we detected a high amount of urea in the blood serum (Table 1).

#### 3.2.3. Macroscopic and Morphometric Examination of Internal Organs

Post mortem examination was performed on the euthanized animals after the experiment in order to evaluate the internal organs and cavities condition. The chest organs and the peritoneal cavity were normally configured and anatomically situated in the test and the control groups. The parietal and visceral layers of the pleura and abdominal membrane were thin, smooth and shiny. The tracheal and bronchial lumina were unrestricted. The aortic intima was smooth and shiny, whitish in color. The chest and the peritoneal cavity contained no free liquid; the peritoneal cavity was also clean, shiny and pink in color.

In the case of every animal, the liver was normally configured and of normal size. In each case, the liver surface was dark red, smooth and uniform, and the capsule was thin and clear. On section, the parenchyma was full-blooded, of medium density.

The kidneys were normally configured and of the normal size. The kidney surfaces were smooth and had a red-brown color, and the capsule was thin, clear and stripped easily. On section, the cortical-medullary junction was normal. The renal pelvises were free.

The spleen was normally configured, dark cherry-colored, of medium density; the capsule was wrinkled. On section, small gray-colored follicles were seen against the dark-red background.

The heart was normally configured and of normal size. On section, the heart muscle revealed a uniform brownish surface, and the muscle was of medium density. The left ventricle was contracted, and the right ventricle contained dark-red clots and liquid blood. The heart valves were thin, shiny and smooth, revealing no abnormalities.

The testes were oval-shaped, of normal size and light gray-colored, and the blood vessels were moderately defined.

The major organs were weighed and the mass coefficients were calculated.

The obtained results showed a tendency of the liver weight to decrease in all experimental groups as well as a significant decrease in the liver mass in the 4–7.5 mg/kg groups (*p* < 0.01), which might be a sign of the organ degeneration caused by the toxic doses of AgNPs-BAC (Table 2).

#### 3.2.4. Histological Examination

Histological examination of the samples of the liver and jejunum fragments in the control and in all experimental groups revealed no pathologies. The hepatocyte cytoplasm of the liver had a normal structure, no dystrophy signs were revealed. In the groups receiving the 5 mg/kg and 7.5 mg/kg doses of AgNPs-BAC, isolated infiltrates consisting of lymphocytes, macrophages and neutrophilic granulocytes were observed, but no statistically significant deviations from the control have been recorded.

#### 3.2.5. Subacute Experiment

**Figure 4:**
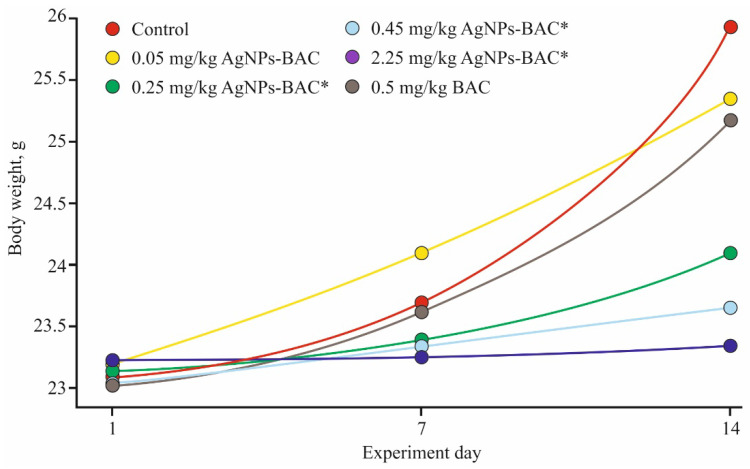
Weight gain dynamics in the subacute experiment. * *p* < 0.01.

AgNPs-BAC and BAC administration in all six experimental groups, as well as in the control group, had no influence upon the general condition and behavior of the animals. The mice in all six groups remained agile and looked well-groomed, and the amounts of food and water consumed were similar to those in the control group. In all tested groups, the animals gained weight (Figure 4), though among the mice receiving the 0.25–2.25 mg/kg doses of AgNPs-BAC, the body weight gain rate was significantly (*p* > 0.05) lower than that of the control. All through the experiment, neither lethal cases, nor signs of intoxication were registered.

#### 3.2.6. Biochemical and Hematological Examination

Table 3 represents the results of the biochemical blood serum test performed after fourteen days of exposure. The results showed that only the 2.25 mg/kg AgNPs-BAC group demonstrated a significant deviation from the control. In this group, we have detected statistically significant differences in the AST and ALT activities which is indicative of hepatosis.

Table 4 shows the morphological and biochemical parameters of the mice blood. The results reveal no significant deviation from the control. The leukograms (Table 4) in all groups show little difference in values between the groups; most variations can be found in the granulocytes amount. The relative content of neutrophils increased. Nevertheless, there is no significant difference between the experimental groups and the control group in the degree of the granulocytes relative content.

In general, the macroscopic pictures of the organs and tissues in all groups, including the control, were similar. The macroscopic examination revealed no expressed defects of the internal organs; every animal had healthy skin and coat. In the pleural cavity, the parietal and visceral layers of the pleura and the pleural organs were unremarkable. The serous membranes of all animals were smooth and shiny. The lungs were pale pink in color, well unfolded and airy, and no indurations or lesions were detected. The hearts of the animals were of the normal size without any signs of ischemia or hypertrophia. The aorta and pulmonary arteries were smooth, without abnormalities or aneurysms. The heart cavities contained a small amount of liquid blood. The myocardium was brown and unremarkable, the tissue turgor was normal. The stomach and pancreas had no conspicuous changes. The liver was normally configured, soft, with a smooth surface. The Glisson’s capsule was thin, clear and relaxed. On section, the hystoarchitectural tissue organization revealed no abnormalities. The kidneys were normally configured and of the normal size, brown-colored and fairly firm. On section, the cortical-medullary junction was normal. The renal pelvises were free. The thymus, thyroid and adrenal glands had no macroscopic differences from the control. The brain was pale gray in color, and had a naturally wet surface, with no detectable edema signs. The meninx vasculosa was closely adherent to the brain matter, and in some areas a moderate dilation and congestion of the venules and small veins was observed. The cerebral ventricles were of the normal size and contained a medium amount of clear colorless fluid.

**Figure 5:**
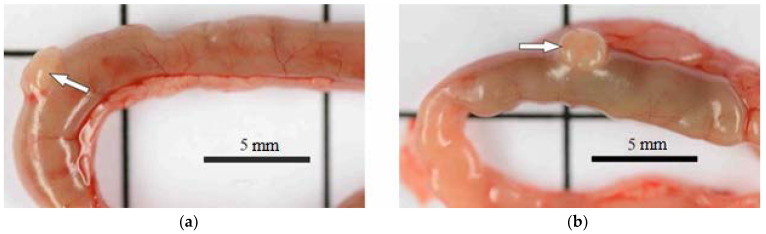
Jejunum area (**a**) control; (**b**) 2.25 mg/kg dose of AgNPs-BAC. Peyer’s patches are indicated by arrows: normal (**a**) and enlarged (**b**).

However, the state of the intestine varied. In the groups exposed to the 0.5 mg/kg dose of BAC and to the 0.05–0.45 mg/kg doses of AgNPs-BAC, the small and large intestine loops had no macroscopic pathologies, while in the group receiving the 2.25 mg/kg dose of AgNPs-BAC, the intestine of some animals revealed small areas of hyperemia and Peyer’s patches were enlarged (Figure 5).

AgNPs-BAC administered at the doses of 0.05 mg/kg and 0.25 mg/kg had no significant effect on the weight coefficients. At the same time, the spleen and liver of the animals exposed to the 0.45 mg/kg and 2.25 mg/kg doses of AgNPs-BAC were smaller than those of the other groups and of the control (Table 5).

#### 3.2.7. Histological Examination

**Figure 6:**
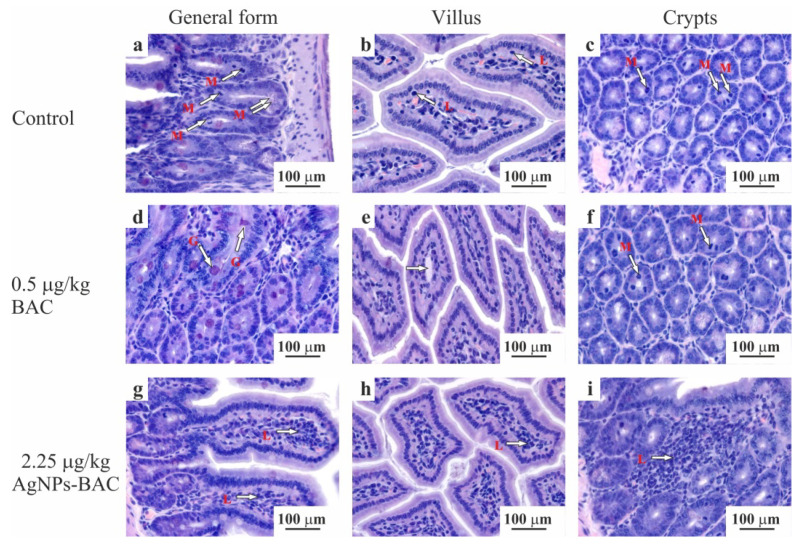
Jejunum histology section of the animals from the control (**a**–**c**) and experimental (**d**–**i**) groups. The micrographs show a general form (**a**,**d**,**g**), the structure of the villus (**b**,**e**,**h**) and crypts (**c**,**f**,**i**). Hematoxylin-eosin staining, ×10 magnification. M—mitotic phase cells (in ana-, meta- and telophase), L—lymphocytes, G—goblet cells mucus.

Histological examination of the intestine revealed no significant differences between the control group and the groups exposed to the 0.5 mg/kg dose of BAC and the 0.05–0.45 mg/kg doses of AgNPs-BAC in terms of the villi size and shape. No signs of the red blood cells degeneration, such as cytoplasmic vacuolization, cell flattening, nuclear pyknosis or nuclear atypia, were detected. We found no inflammatory changes of the villi (e.g., hemorrhage, epithalaxia, adhesions between the neighbouring villi tips, etc.) (Figure 6a–f). In the 2.25 mg/kg AgNPs-BAC group, the following peculiarities of the intestine formation were observed: the columnar enterocytes possessed elongated nuclei and were situated closer to each other compared to the control, forming in some areas fence-like structures (Figure 6g). There were some cases of the brush border structure disruption in the columnar enterocytes where the structure could not be clearly observed in some areas of the villus (Figure 6h). The number of goblet cells in the epithelium of the villi and of the crypts increased. The goblet cells mucus was stained less intensively compared to the control. The crypt lumina were dilated and contained a large amount of mucus. The number of mitotic figures in the crypts increased (Figure 6i). We could see individual lymphocytic infiltrates; the examination showed a small increase in the villi lateral dimensions caused by their hyperemia, swelling and a moderate lymphocytic infiltration of the stroma (Figure 6h). The space between the villi was filled with the substance stained similar to the goblet cells mucus.

Histological examination of the liver of the animals from the control group, the 0.5 mg/kg BAC and the 0.05–0.45 mg/kg AgNPs-BAC groups revealed no critical differences. In the liver of some animals from the 0.45 mg/kg AgNPs-BAC group, we discovered a small number of infiltrates <100 μm in diameter consisting of lymphocytes, neutrophilic granulocytes and macrophages. The adjoining hepatocytes were characterized by more intensive staining of the oxyphilic cytoplasm. Neither in the infiltrates, nor in the adjoining regions did we detect hepatocytes with the signs of necrosis.

In the 2.25 mg/kg AgNPs-BAC group, the staining of the cytoplasm of hepatocytes was nonuniform. In the cytoplasm, we could identify numerous lighter stained areas with uneven edges. In a number of cases, the cytoplasm showed signs of grainy degeneration. In some cases, the cavities with ill-defined edges occupied almost the entire cytoplasm. The abnormally structured hepatocytes were mainly identified near the portal triads of the lobules. A certain number of hepatocyte nuclei were malformed and vacuolated; in some cases, the nuclei contained homogenous oxyphilic masses while the chromatin was unevenly dispersed and pushed closely with the nuclear membrane. In parenchyma, we identified infiltrates consisting of lymphocytes, neutrophilic granulocytes and macrophages. In the adjoining areas, hepatocytes with darker stained oxyphilic cytoplasm were observed.

**Figure 7:**
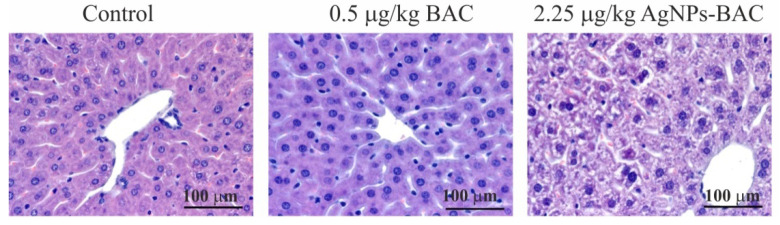
Jejunum histology section of the animals from the experimental and control groups. Hematoxylin-eosin staining, ×10 magnification. M—mitotic phase cells (in ana-, meta- and telophase), L—lymphocytes, G—goblet cells mucus.

Upon examination, we detected no evidence of necrosis. We found no silver deposits in the liver macrophages (Figure 7).

As mentioned above, no accumulation of silver in the organs of laboratory animals was detected using optical microscopy. In our previous studies, we observed that less than 0.088 mg/kg of silver were accumulated in the liver of broiler chickens following oral administration of a dispersion containing 2 μg/mL of colloidal silver stabilized with BAC at a daily dose of 1 mL per 1 kg of body weight for 45 days [56]; however, no negative effects of nanosilver have been detected. In another case, colloidal silver stabilized with BAC was introduced daily at a dose of 0.32 μg per 1 kg of body weight, with almost all silver remaining in the bird’s body, while the increase of the daily administered dose to 1.92 μg per 1 kg of body weight resulted in only 30% of the silver remaining in the body [57]. At the same time, the accumulation of silver in the organs did not affect the body weight or increase dynamics negatively.

Data obtained by other authors show that AgNPs administered orally are accumulated in the organs of laboratory animals, especially the liver. For example, this was the case following oral administration of 56 nm AgNPs at a dosage of 30–500 mg/kg for 90 days to male and female F344 rats [11]. At the end of a similar period of exposure of male Sprague Dawley rats to 15 and 20 nm AgNPs at a dosage of 90 mg/kg, silver accumulation was also observed mainly in the liver and spleen of animals [58]. Oral administration of AgNPs of three sizes (10 nm, 75 nm and 110 nm) to Sprague Dawley rats at a dosage of 9–36 mg/kg for 13 weeks led to size-dependent accumulation of silver in the kidneys, spleen and liver [18]. 92 nm AgNPs administered to male Wistar rats at a dosage of 0.5 mg/kg for 45 days caused the accumulation of silver in the blood, liver and kidneys [59].

Thus, based on our previous results and numerous data from other researchers, we assume that silver is usually accumulated in internal organs of animals and directly causes histopathological effects.

## 4. Discussion

New areas of application of nanoparticles appear every year, although the question of their safety has not yet been resolved, and the number of papers providing evidence of their toxicity to healthy animal cells is ever-increasing. Based on numerous toxicological studies performed on laboratory animals, the minimum AgNPs dose inducing the first signs of intoxication appears to be 1 mg/kg [60].

### 4.1. Acute Experiment

The results of our own study showed that in the acute experiment, the AgNPs-BAC toxicity level is consistent with the previously reported data. In all experimental groups the weight gain rate differed from that of the control. In particular, in the groups exposed to the doses of 0.8–1.5 mg/kg of AgNPs-BAC, the negative dynamic was observed, which is probably due to the adverse effect on the gut microbiome [61] or to the AgNPs-BAC penetrating through the intestinal wall and into the internal milieu, which correlates with the general impact on the animal’s health. In the groups exposed to the larger doses (4 mg/kg, 5 mg/kg and 7.5 mg/kg), the picture was somewhat different, i.e., during the first 7 days, the weight gain dynamic was negative, similar to the lower-dosage groups, which agrees with the results reported by other research teams rather well [62]. Nevertheless, during the second week, we observed a positive dynamic. This supposedly happened due to the fact that higher concentrations of NPs have an increased aggregation rate helping the protective system of the organism recognize them and remove them from the body more effectively [63,64], thus minimizing their negative impact on the gut microflora.

At the same time, the authors [65] showed that big doses of AgNPs can cause adiposis, with the fat tissue gain rate increasing significantly starting from the seventh day after the AgNPs administration. It might be so that the same effect was observed following the administration of the 4–7.5 mg/kg AgNPs-BAC dose (Figure 2).

Exposure to the dose of 0.8–4 mg/kg of AgNPs-BAC had no adverse effects on the homeostasis and hematopoietic system and produced no significant alterations in the biochemical parameters. At the same time, in the groups receiving the 5 mg/kg and 7.5 mg/kg doses we registered an increase in the aminotransferase content in the blood serum due to hepatolysis or hepatitis caused by the studied material. The tests also revealed a shift in the albumin–globulin ratio resulting either from the excessive consumption of albumin as the primary toxin-binding protein, or from the increase in the globulins production caused by inflammation. The increased protein utilization was also revealed as we detected a high urea amount in the blood serum (Table 3).

Due to the fact that the gastrointestinal tract and liver are the primary targets of orally administered toxic substances [66], particular attention was paid to these organs.

Based on the calculation of the mass coefficients of the internal organs, a tendency of the liver weight to decrease was revealed in all experimental groups and a significant decrease in the liver mass in the 4–7.5 mg/kg groups (*p* < 0.01) was observed, which might be a sign of the organ degeneration caused by the toxic doses of AgNPs-BAC. This effect might be related to the fact that the liver receives the highest amount of AgNPs circulating in the bloodstream [67,68,69]. Though the histological examination shows that none of the studied AgNP-BAC doses produced a statistically significant effect upon the mice liver, we can assume that the toxic effects were present in the groups exposed to the 4–7.5 mg/kg dose of AgNP-BAC as evidenced by the discovery of isolated infiltrates consisting of lymphocytes, macrophages and neutrophilic granulocytes. Drawing a comparison between our results and the data from the previous research in terms of concentrations and potential toxicity proved challenging because of the differences in the employed experimental schemes, although the absence of observable pathological changes in the liver following single administration of small doses of AgNPs (5 mg/kg) has been confirmed in [67], while for rabbits the same dose caused formation of inflammatory infiltrates in the liver [68].

### 4.2. Subacute Experiment

AgNPs-BAC and BAC administration in all six experimental groups had no influence on the general condition and behavior of the animals. The mice in all six groups remained agile, had well-groomed coats, and the amounts of food and water consumed were similar to those of the control group. All through the experiment, neither lethal cases nor signs of intoxication were registered. In all tested groups, the animals gained weight, although among the mice receiving the dose of 0.25–2.25 mg/kg of AgNPs-BAC, the body weight gain rate was significantly (*p* > 0.05) below that of the control group. The obtained data differs from the results of similar studies where intraperitoneal administration of AgNPs at the doses of 1, 2 and 4 mg/kg [27] and oral administration of the same material at the doses of 2.25, 4.5 and 9 mg/kg [70] during the same period of time resulted in no alterations of the studied parameter. The slower rate of the body weight gain might be attributed to the general irregularities in the gastrointestinal tract functions under the influence of the orally administered AgNPs-BAC and to the development of inflammatory processes in the intestines.

Biochemical examination of the blood serum revealed no significant deviations from the control group except in the group exposed to the 2.25 mg/kg dose of AgNPs-BAC. In this group, we have detected differences in the activity of both transaminases which is an evidence of hepatosis. Significantly reduced values of the ALT (by 76.9%) and AST (by 95.5%) in the serum might be indicative of a decrease in the functioning hepatocytes mass and disruption in the regenerative functions of the liver. These data do not agree with the results of the toxicological studies of non-functionalized AgNPs where the studied parameters increased after oral administration of the material at the doses of 20 mg/kg and below [31]. The authors connect this increase to the free radicals released when the hepatocytes were damaged. A similar decrease in the ALT values has been observed by another research team, but at much higher doses (300 and 1000 mg/kg) [71]. The mass coefficients of the liver showed a decrease in the studied parameter compared to the control group, in a manner similar to the acute experiment, which indicates moderate hepatotoxic activity of AgNPs-BAC.

Previous research has revealed a high probability of the hepatotoxicity development under the influence of AgNPs [11,72,73]. The results of the present study are in partial conformity with some earlier findings describing the liver as the target organ for AgNPs. Histological examinations of the liver of the animals from the control, the 0.5 mg/kg BAC and the 0.05–0.45 mg/kg AgNPs-BAC groups revealed no critical differences. In the liver of some animals from the 0.45 mg/kg AgNPs-BAC group, we discovered a small number of infiltrates <100 μm in diameter consisting of lymphocytes, neutrophilic granulocytes and macrophages. The adjoining hepatocytes were characterized by more intensive staining of the oxyphilic cytoplasm. Neither in the infiltrates, nor in the adjoining regions did we detect hepatocytes with the signs of necrosis.

In the 2.25 mg/kg AgNPs-BAC group, the staining of the cytoplasm of hepatocytes was non-uniform. In the cytoplasm, we could identify numerous lighter areas with uneven edges. In a number of cases, the cytoplasm showed signs of grainy degeneration. In some cases, the cavities with ill-defined edges occupied almost the entire cytoplasm. The abnormally structured hepatocytes were mainly identified near the portal triads of the lobules. A certain number of hepatocyte nuclei were malformed and vacuolated; in some cases, the nuclei contained homogenous oxyphilic masses, and the chromatin was unevenly dispersed and pushed closely with the nuclear membrane. In parenchyma, we identified infiltrates consisting of lymphocytes, neutrophilic granulocytes and macrophages. In the adjoining areas, hepatocytes with darker stained oxyphilic cytoplasm were observed.

In the groups exposed to the 0.45 and 2.25 mg/kg doses of AgNPs-BAC, the mass coefficients of the spleen were reduced by over 50 percent. The results agree with the previous studies well [70].

Macroscopic examination of the intestine showed that in the group receiving the 2.25 mg/kg dose of AgNPs-BAC, the intestine of some mice had small areas of hyperemia and Peyer’s patches were enlarged. These findings confirm our assumptions about the reason of the slow body weight gain rate of the experimental animals.

Histological examinations of the intestine revealed no significant differences between the control group and the groups exposed to the 0.5 mg/kg dose of BAC and the 0.05–0.45 mg/kg dose of AgNPs-BAC. In the 2.25 mg/kg AgNPs-BAC group, the following peculiarities of intestine formation were observed: the columnar enterocytes possessed elongated nuclei and were situated closer to each other compared to the control group, forming in some areas fence-like structures. There were several cases of the brush border structure disruption in the columnar enterocytes. The number of goblet cells was increased in the epithelium of the villi and of the crypts. The goblet cells mucus was stained less intensively compared to the control group. The crypt lumina were dilated and contained a large amount of mucus. The number of mitotic figures in the crypts increased. We could see individual lymphocytic infiltrates; a small increase of the lateral villi dimensions caused by their hyperemia was also detected, together with swelling and a moderate lymphocytic infiltration of the stroma. The space between the villi was filled with the substance stained similar to the goblet cells mucus. Such changes in the intestine structure are consistent with the results obtained during the course of other studies of the AgNPs toxicity [24].

Thus, our results suggest that AgNPs-BAC combine high biological activity with the absence of pronounced entero- and hepatotoxicity if administered orally once at the doses less than 4 mg/kg or repeatedly at the doses less than 0.45 mg/kg. Nanoparticles seem to produce diverse effects, simultaneously inhibiting growth of the pathogenic intestinal flora and damaging the animal organs due to the inherent toxicity.

Oral method of administration brings the NPs in immediate contact with the gastrointestinal tract. It is possible that NPs: (i) are accumulated in the mucous layer and either get quickly removed from the organism with the mucous layer renewal, or penetrate the deeper layers of the intestinal wall, remaining in the organism much longer; (ii) display their bactericidal properties by affecting the intestinal microbiota; (iii) interact with the intestinal epithelial cells causing local inflammation, (iv) penetrate the intestinal lymphatic tissue affecting the intestinal immunity; and (v) through the intestinal epithelium reach the bloodstream and affect other organs, the liver in particular [74]. The mechanisms of further AgNPs action in the organism depend on the type of interaction between the nanoparticles and the gastrointestinal tract. For example, in the bloodstream, the AgNPs become parts of protein complexes and get excreted by the liver and kidneys. Induction of metallothioneins, the proteins that bind silver, significantly affects metabolism of the metal. This reduces the cellular toxicity of silver and stimulates further tissue regeneration. The AgNP-linked toxicity mechanisms are not unidirectional: they can combine simultaneous development of several pathological processes [66,75]. AgNPs can enter cells and produce reactive oxygen species, thus causing oxidative damage to the cell membranes, organelles (lysosomes, mitochondria) and nucleus and leading the entire cell to apoptosis or necrosis. [66]. AgNPs can also be a source of Ag+ ions. The ions penetrate the cell walls and interact with the cell membranes, thus changing their structure and function, and causing extravasation of the cell content [76]. Disruption of the genetic information replication, including the synthesis of DNA, RNA and mRNA, might also lead to cell death. Nevertheless, further studies are required in order to obtain a better understanding of all toxicity mechanisms of such particles. Meanwhile, the dose, exposure time, size, shape, surface functionalization, surface charge and method of administration of AgNPs remain the most important factors affecting severity of the arising pathological processes. It can be assumed that modification of AgNPs with BAC increases their biocompatibility upon enteral administration which can be applied in the biomedical and veterinary fields.

## 5. Conclusions

The study confirms that no significant entero- and hepatotoxic effects following a single exposure to doses of AgNPs-BAC smaller than 4 mg/kg were detected. Repeated exposure to the doses of AgNPs-BAC below 0.45 mg/kg and to the doses of BAC below 0.5 mg/kg upon enteral administration also led to no adverse effects. In the course of the acute experiment, the higher AgNPs-BAC dose resulted in increased quantities of aminotransferases and urea, as well as an albumin–globulin ratio shift, which are indicative of inflammatory processes. Besides, the relative mass of the liver of mice was smaller compared to the control. During the subacute experiment, the groups treated with the 0.25–2.25 mg/kg doses of AgNPs-BAC had a lower weight gain rate compared to the control, while the groups treated with the 2.25 mg/kg dose of AgNPs-BAC showed statistically significant variations in the blood serum transaminases activity, which indicated hepatosis. It should be noted that the spleen and liver of the animals from the groups treated with the 0.45 mg/kg and 2.25 mg/kg doses of AgNPs-BAC were more than two times smaller compared to those of the control. In the intestines of some animals from the group treated with the 2.25 mg/kg dose of AgNPs-BAC, small areas of hyperemia and enlarged Peyer’s patches were observed. Histological examination confirmed the initial stages of the liver and intestinal wall inflammation.

It should be mentioned that throughout the experiments, we registered neither fatalities, nor visible signs of intoxication. In most cases, the AgNPs-BAC doses displayed no significant entero- and hepatotoxicity, which is likely due to the effects of stabilization with the non-toxic BAC. Thus, modification of silver nanoparticles with benzyldimethyl[3-myristoylamine)-propyl]ammonium chloride monohydrate increases their biocompatibility upon enteral administration that can be useful in terms of biomedical and veterinary application.

## Figures and Tables

**Table 1:** Morphological and biochemical parameters of mice blood following single administration of high doses of AgNPs stabilized with benzyldimethyl[3-myristoylamine)-propyl]ammonium chloride monohydrate (AgNPs-BAC).

Parameters/Group	5 mg/kg Dose of AgNPs-BAC	7.5 mg/kg Dose of AgNPs-BAC	5 mg/kg Dose of BAC	Control
Red blood cells, ×1012	7.84 ± 0.48	7.24 ± 0.38	7.12± 0.39	6.95 ± 0.41
White blood cells, ×109	3.74 ± 0.36	5.15 ± 0.64	5.17 ± 0.38	5.2 ± 0.59
Haemoglobin, g/L	128.07 ± 5.27	130.78 ± 8.26	127.46 ± 4.33	126.33 ± 3.23
White blood cell differential				
Neutrophils:				
immature	2.7 ± 0.75	1.2 ± 0.38	1.3 ± 0.52	1.16 ± 0.65
band	3.2 ± 0.43	3.9 ± 0.54	3.3 ± 1.2	4.1 ± 1.1
segmented	27.55 ± 2.3	16.4 ± 2.25	21.79 ± 2.8	21.33 ± 4.7
Eosinophils	0	0.6 ± 0.06	0.1 ± 0.01	0.1 ± 0.01
Basophils	0	0	0	0
Monocytes	0	0	0	0
Lymphocyte	66.55 ± 1.79	78.0 ± 2.18	76.0 ± 3.29	75.0 ± 5.05
Aspartate transaminase, IU/L	346.37 ± 7.1 *	361.75 ± 4.63 *	322.88 ± 5.78	319.83 ± 8.76
Alanine transaminase, IU/L	103.9 ± 0.55 *	125.9 ± 0.53 *	87.12 ± 0.94	87.12 ± 0.94
Total protein, g/L	54.71 ± 7.77	63.22 ± 2.68	56.32 ± 2.36	53.75 ± 1.56
Albumin, g/L	19.53 ± 0.88	23.83 ± 1.24	22.75 ± 0.76	22.05 ± 0.78
Globulins, g/L	35.48 ± 2.03 *	37.9 ± 2.33 *	32.44 ± 1.11	30.45 ± 1.12
Albumin/Globulin	0.56 ± 0.05 *	0.66 ± 0.07 *	0.7 ± 0.03	0.72 ± 0.03
Urea, mmol/L	12.05 ± 0.26 *	14.62 ± 0.71 *	9.2 ± 0.37	8.3 ± 0.35
Creatinine, μmol/L	38.75 ± 2.33	73.42 ± 8.13	55.53 ± 3.27	50.63 ± 5.24

Note: A/G stands for albumin/globulin ratio. * *p* < 0.05.

**Table 2:** The mass coefficients of the mice organs following single AgNPs-BAC administration.

Group	Heart, %	Liver, %	Spleen, %	Kidney, %
Control	0.39 ± 0.03	6.22 ± 0.2	0.89 ± 0.16	0.77 ± 0.02
5 mg/kg dose of BAC	0.4 ± 0.02	6.03 ± 0.17	0.91 ± 0.15	0.79 ± 0.03
0.8 mg/kg dose of AgNPs-BAC	0.41 ± 0.02	5.53 ± 0.27	0.67 ± 0.13	0.84 ± 0.07
1 mg/kg dose of AgNPs-BAC	0.41 ± 0.01	5.73 ± 0.17	0.67 ± 0.09	0.79 ± 0.1
1.2 mg/kg dose of AgNPs-BAC	0.41 ± 0.02	5.58 ± 0.23	0.65 ± 0.11	0.82 ± 0.07
1.5 mg/kg dose of AgNPs-BAC	0.42 ± 0.02	5.55 ± 0.19	0.65 ± 0.09	0.84 ± 0.1
4 mg/kg dose of AgNPs-BAC	0.42 ± 0.03	4.92 ± 0.22 *	0.93 ± 0.06	0.86 ± 0.03
5 mg/kg dose of AgNPs-BAC	0.41 ± 0.03	4.91 ± 0.19 *	0.89 ± 0.07	0.81 ± 0.03
7.5 mg/kg dose of AgNPs-BAC	0.42 ± 0.02	4.94 ± 0.19 *	0.92 ± 0.07	0.86 ± 0.03

Note: * *p* < 0.01 compared to control.

**Table 3:** Biochemical examination results (M ± m).

Group	Total Protein, g/L	Albumin, g/L	Urea, mmol/L	Creatinine, μmol/L	ALT, UI/L	AST, UI/L
Control	53.75 ± 1.26	22.05 ± 0.78	8.3 ± 0.35	50.63 ± 5.24	38.9 ± 1.6	66.56 ± 11.5
0.5 mg/kg dose of BAC	52.63 ± 1.26	24.05 ± 0.63	8.41 ± 0.41	55.62 ± 6.21	39.9 ± 1.7	65.12 ± 10.3
0.05 mg/kg dose of AgNPs-BAC	51.8 ± 1.7	27.7 ± 1.4	8.3 ± 0.6	67.12 ± 8.04	37.2 ± 1.4	64.7 ± 1.8
0.45 mg/kg dose of AgNPs-BAC	52.0 ± 1.6	29.2 ± 1.1	8.5 ± 0.5	51.54 ± 6.45	40.4 ± 2.0	53.3 ± 1.7
0.25 mg/kg dose of AgNPs-BAC	53.2 ± 1.9	28.7 ± 1.2	8.6 ± 0.5	52.42 ± 3.12	39.5 ± 1.8	64.1 ± 1.6
2.25 mg/kg dose of AgNPs-BAC	51.65 ± 1.97	29.72 ± 1.3	8.74 ± 0.47	80.33 ± 18.02	9 ± 0.14 *	3 ± 0.52 *

* Statistically significant deviation from the control (*p* < 0.05).

**Table 4:** Morphological and biochemical parameters of the mice blood after multiple intragastric AgNPs-BAC administration.

	0.05 mg/kg Dose of AgNPs-BAC	0.45 mg/kg Dose of AgNPs-BAC	0.25 mg/kg Dose of AgNPs-BAC	2.25 mg/kg Dose of AgNPs-BAC	Control	0.5 mg/kg Dose of BAC
Red blood cells, ×10^12^	6.96 ± 0.35	7.08 ± 0.49	6.98 ± 0.34	7.13 ± 0.29	6.95 ± 0.41	6.97 ± 0.36
White blood cells, ×10^9^	5.2 ± 0.36	3.98 ± 0.2	5.01 ± 0.35	3.67 ± 0.29	5.2 ± 0.59	4.8 ± 0.44
Platelets, 10^3^/μL	734 ± 60	725 ± 62	736 ± 58	741 ± 44	732 ± 56	734 ± 57
Haemoglobin, g/L	128.92 ± 6.33	130.14 ± 3.48	129.01 ± 5.76	134.62 ± 4.77	126.33 ± 3.23	129.43 ± 3.11
White blood cell differential, %						
Neutrophils:						
immature	1.7 ± 0.75	1.6 ± 0.53	1.2 ± 0.38	1.36 ± 0.43	1.16 ± 0.65	1.3 ± 0.45
band	3.2 ± 0.43	3.2 ± 0.52	3.9 ± 0.54	2 ± 0.52	4.1 ± 1.1	3.6 ± 0.6
segmented	22.55 ± 2.3	22.15 ± 2.14	22.4 ± 2.25	22.09 ± 3.24	21.33 ± 4.7	22.16 ± 4.3
Eosinophils	0	0.36 ± 0.05	0.36 ± 0.06	0.36 ± 0.05	0.1 ± 0.01	0
Basophils	0	0	0	0	0	0
Monocytes	0	0	0	0	0	0
Lymphocyte	76.5 ± 1.79	74.31 ± 1.38	74.0 ± 2.18	74 ± 3.29	75.0 ± 5.05	73.0 ± 4.6

No statistically significant deviations from the control were observed.

**Table 5:** The mass coefficients of the internal mice organs upon multiple intragastric administration of the studied substances (M ± m, g).

Organ	Control	0.05 mg/kg Dose of AgNPs-BAC	0.25 mg/kg Dose of AgNPs-BAC	0.45 mg/kg Dose of AgNPs-BAC	2.25 mg/kg Dose of AgNPs-BAC	0.5 mg/kg Dose of BAC
Kidneys,%	0.77 ± 0.02	0.77 ± 0.03	0.78 ± 0.05	0.7 ± 0.07	0.71 ± 0.02	0.79 ± 0.03
Liver,%	6.22 ± 0.2	6.5 ± 0.39	5.86 ± 0.12	5.1 ± 0.26 *	4.4 ± 0.09 *	6.15 ± 0.12
Heart,%	0.39 ± 0.03	0.43 ± 0.03	0.37 ± 0.08	0.42 ± 0.02	0.42 ± 0.007	0.38 ± 0.08
Spleen,%	0.89 ± 0.16	0.65 ± 0.11	0.55 ± 0.4	0.46 ± 0.07 *	0.41 ± 0.03 *	0.82 ± 0.11

* Statistically significant deviation from the control (*p* < 0.05).

## Data Availability

Data is contained within the article.
